# MYT3, A Myb-Like Transcription Factor, Affects Fungal Development and Pathogenicity of *Fusarium graminearum*

**DOI:** 10.1371/journal.pone.0094359

**Published:** 2014-04-10

**Authors:** Yongsoo Kim, Hun Kim, Hokyoung Son, Gyung Ja Choi, Jin-Cheol Kim, Yin-Won Lee

**Affiliations:** 1 Department of Agricultural Biotechnology and Center for Fungal Pathogenesis, Seoul National University, Seoul, Republic of Korea; 2 Eco-friendly New Materials Research Group, Research Center for Biobased Chemistry, Division of Convergence Chemistry, Korea Research Institute of Chemical Technology, Daejeon, Republic of Korea; University of Wisconsin – Madison, United States of America

## Abstract

We previously characterized members of the Myb protein family, MYT1 and MYT2, in *Fusarium graminearum*. MYT1 and MYT2 are involved in female fertility and perithecium size, respectively. To expand knowledge of Myb proteins in *F. graminearum*, in this study, we characterized the functions of the *MYT3* gene, which encodes a putative Myb-like transcription factor containing two Myb DNA-binding domains and is conserved in the subphylum Pezizomycotina of Ascomycota. MYT3 proteins were localized in nuclei during most developmental stages, suggesting the role of MYT3 as a transcriptional regulator. Deletion of *MYT3* resulted in impairment of conidiation, germination, and vegetative growth compared to the wild type, whereas complementation of *MYT3* restored the wild-type phenotype. Additionally, the Δ*myt3* strain grew poorly on nitrogen-limited media; however, the mutant grew robustly on minimal media supplemented with ammonium. Moreover, expression level of nitrate reductase gene in the Δ*myt3* strain was decreased in comparison to the wild type and complemented strain. On flowering wheat heads, the Δ*myt3* strain exhibited reduced pathogenicity, which corresponded with significant reductions in trichothecene production and transcript levels of trichothecene biosynthetic genes. When the mutant was selfed, mated as a female, or mated as a male for sexual development, perithecia were not observed on the cultures, indicating that the Δ*myt3* strain lost both male and female fertility. Taken together, these results demonstrate that MYT3 is required for pathogenesis and sexual development in *F. graminearum*, and will provide a robust foundation to establish the regulatory networks for all Myb-like proteins in *F. graminearum*.

## Introduction

The homothallic ascomycete fungus *Fusarium graminearum* is a prominent plant pathogen that causes Fusarium head blight (FHB) on cereal crops and ear rot on maize [1], [2], resulting in a severe reduction of crop yield and production of mycotoxins (e.g., trichothecenes and zearalenone) that are dangerous to animals and humans [3]. In particular, sexual spores (ascospores) of *F. graminearum* are believed to be the primary inocula for FHB epidemics in cereal crops [4]. The spores overwinter within a fruiting body (perithecium) on plant debris, and are forcibly discharged into the air under milder temperatures and moderate moisture [4], [5]. To date, several genes related to sexual development have been studied in *F. graminearum*, and those genes are known to be closely related to disease development [6], [7], [8], [9], [10].

In the *F. graminearum* genome, 19 transcription factors encoding Myb-like proteins were identified at a previous work [11]. Of these Myb-like proteins, we found that MYT1 and MYT2 transcription factors are involved in female fertility and perithecium size, respectively [12], [13]. Furthermore, several deletion mutants of putative Myb transcription factors, including FGSG_01915, FGSG_02719, and FGSG_12781, in *F. graminearum* exhibited abnormality in sexual development [11]. These data suggest that the Myb transcription factors are required for normal sexual development of *F. graminearum*.

Transcription factors that contain the Myb DNA-binding domain are involved in various cellular processes in eukaryotes, including cell proliferation, apoptosis, differentiation, metabolism, and stress responses [14], [15], [16], [17]. The v-Myb DNA-binding domain was originally identified from avian myeloblastosis virus (AMV) as a truncated version of a cellular progenitor c-Myb that contains three Myb DNA-binding domains, which are referred to as R1, R2, and R3 [18], [19]. The Myb DNA-binding domains identified from vertebrates, plants, and oomycetes are relatively conserved between species, and most Myb proteins contain one to three variants of the Myb DNA-binding domain, such as R1, R2R3, and R1R2R3 [14], [20], [21]. Each Myb DNA-binding domain spans approximately 52 amino acids arranged as tandem repeats of a helix-turn-helix motif [22], [23]. This repeat region is involved in DNA-binding, and the specificity of DNA binding is known to be dependent on the third α-helices of R2 and R3 [24].

Currently, a few Myb-like proteins have been identified and characterized in fungal species. Ag*BAS1,* a homolog of *Saccharomyces cerevisiae BAS1*, was identified in *Ashbya gossypii*, and the deletion mutant of Ag*BAS1* exhibited auxotrophy for adenine and a delay in spore germination [25]. Furthermore, AgBAS1 controls the adenine-mediated regulation of the purine biosynthetic pathway by regulating the gene expression of *ADE4* by binding to the *ADE4* promoter [25]. In *Aspergillus nidulans*, a Myb transcription factor, FlbD, controls asexual and sexual differentiation [26], [27]. The *flbD* mutants were unable to develop the cleistothecial peridium, a specialized external tissue that differentiates during fruiting body formation, and exhibited severely defective conidiation. In addition, a Myb-like protein *PsMYB1* of *Phytophthora sojae,* which causes soybean root rot, was identified by differential expression between wild type and MAP kinase mutants, and the *PsMYB1* is known to be involved in zoospore-mediated pathogenicity [28].

In the present report, as a series of MYT1 and MYT2, we aimed to expand the current knowledge of how Myb transcription factors regulate sexual development of *F. graminearum*, with an emphasis on the characterization of the gene involved in the formation of perithecia and ascospores. To this end, we chose to investigate *MYT3,* which encodes a putative Myb-like transcription factor, and further characterized its biological function by molecular approaches. Our results demonstrate that *MYT3* is required for conidiation, vegetative growth, sexual development, and pathogenicity.

## Materials and Methods

### Fungal strains and media

All strains used in this study are listed in Table 1. The wild-type strain Z-3639 and mutants derived from the wild-type strain were maintained on complete media (CM) [29], and stored in 20% glycerol at −80°C. The growth rates of the wild type and transgenic strains were measured in CM and minimal media (MM; 3 g KH_2_PO_4_, 0.5 g MgSO_4_, 0.5 g KCl, 30 g sucrose, and 20 g agar per liter). To observe responses to nitrogen sources, the MM was modified by addition of 2 g NaNO_3_ or 2 g (NH_4_)_2_SO_4_ per liter. Conidia production was induced in carboxymethyl cellulose (CMC) medium [30]. Minimal liquid medium supplemented with 5 mM agmatine (MMA) was used for trichothecene analysis [31].

**Table 1:** *Fusarium graminearum* strains used in this study.

Strain	Genotype	Source or reference
Z-3639	wild type	66
Δ*myt3*	Δ*myt3*::*GEN*	11
HK33	Δ*acl2*::*GEN*; *HYG*-*P_ZEAR_*::ACS2	37
mat1r	Δ*mat1-1*::*GEN*; *hH1*::*RFP-GEN*	41
Δ*mat2*	Δ*mat1-2*::*GFP-HPH*	45
Δ*myt3*-com	Δ*myt3*::*MYT3-HYG*	In this study
*MYT3*-oe	*P_EF1α_*::*MYT3-GEN*	In this study
*MYT3*-gfp	*MYT3*::*GFP-HPH*	In this study
*MYT3*-gr	*MYT3*::*GFP-HPH*; *hH1*::*RFP-GEN*	In this study

### Nucleic acid manipulations and protein sequence analysis

Fungal strains grown in CM broth for 5 days at 25°C were harvested and lyophilized. The procedures for isolation of genomic DNA were performed as described [29]. Standard methods were followed for restriction endonuclease digestion, agarose gel electrophoresis, and Southern blotting [32]. To measure expression level of transcripts, total RNA was extracted by using the Easy-Spin Total RNA Extraction Kit (iNtRON Biotechnology, Seongnam, Korea), and the first strand of cDNA was synthesized from the RNA by SuperScriptIII reverse transcriptase (Invitrogen, Carlsbad, CA, USA). PCR primers were obtained from Bionics (Seoul, Korea), listed in Table S1. Protein sequence alignment was performed by Clustal Omega [33], and drawn by BOXSHADE 3.31 available at http://www.ch.embnet.org/software/BOX_form.html. InterPro (http://www.ebi.ac.uk/interpro/) and Fungal Transcription Factor Database (FTFD; ftfd.snu.ac.kr) were used to search for the Myb DNA-binding domains. Automated mode of SWISS-MODEL [34] and YASPIN [35] were used for the structural analysis of Myb-like DNA binding domains. Nuclear localization sequences (NLSs) were identified by cNLS Mapper [36].

### Complementation and overexpression of *MYT3*

For the complementation of the Δ*myt3* strain, intact copies of *MYT3* (FGSG_00324; *Fusarium* Comparative Sequencing Project at the Broad Institute of MIT and Harvard, http://www.broadinstitute.org) were amplified from *F. graminearum* wild-type Z-3639 with primer pair MYT3-5N/MYT3-3N. The selection marker, hygromycin phosphotransferase (*HPH*), was amplified from the HK33 strain with primer pair HPH-F/HPH-R [37]. These two fragments were co-transformed into the protoplast of the Δ*myt3* strain. For fungal transformations, the methods for the preparation of protoplasts and regeneration of transformants were performed as described previously [38]. Transformants were confirmed by Southern blot analysis. To generate the *MYT3* overexpression strains, the 5′ flanking region and ORF region of *MYT3* were amplified with primer pairs UP-5F/5R-GEN and MYT3-F/MYT3-R, respectively. A PCR fragment with the geneticin resistant gene (*GEN*) and the elongation factor 1α promoter (*P_EF1α_*) was amplified from pSKGEN [37] using primer pair Neo-F/EF-R. These three amplicons were combined as previously described [39]. The resulting product was amplified with nested primers MYT3-5N and MYT3-RN to create a 4.3-kb product containing *GEN* and *P_EF1α_* fused to the *MYT3* ORF. The final construct was transformed into the wild-type strain.

### Cellular localization of MYT3

To investigate cellular localization of MYT3, we generated a strain carrying green fluorescent protein (GFP) fused to C-terminus of MYT3. A PCR fragment including *GFP* and *HPH* was amplified from the pIGPAPA plasmid [40] with primers GFP-F and HYG-F1. The 5′ flanking region, which is the *MYT3* C-terminus without its own terminator sequence, was amplified from the wild-type strain with primers MYT3-5F and MYT3-5R. The 3′ flanking region of *MYT3* was amplified by primers MYT3-3F and MYT3-3R. These three resulting PCR products were fused as described [39]. The resulting PCR product was used as a template with primers MYT3-gfpF and MYT3-gfpR for a final construct. Subsequently, the final PCR products were transformed into the wild-type strain.

To observe co-localization of MYT3 with a nuclear protein, a resulting *MYT3*-gfp transformant was outcrossed with a mat1r strain that contains red fluorescent protein (RFP) fused to histone H1 as generated previously [41]. Strains containing both *MYT3*::*GFP* and *hH1*::*RFP* were selected by antibiotic resistance and confirmed by PCR. Microscopic observation was performed by a DE/Axio Imager A1 microscope (Carl Zeiss, Oberkochen, Germany) with the filter set 38HE (excitation 470/40; emission 525/50) for GFP and the filter set 15 (excitation 546/12; emission 590) for RFP.

### Western blot analysis

Mycelia and perithecia were harvested from carrot agar cultures, and ground by liquid nitrogen. The ground tissues (approximately 100 to 150 mg) were used for the extraction of total protein with 300 μl of extraction buffer [50 mM Tris-Cl, pH 8.4; 192 mM glycine; 0.1% sodium dodecyl sulfate (SDS); 1 mM protease inhibitor phenylmethanesulfonylfluoride]. After centrifugation at 16000 g for 10 min, supernatant was used for the protein analysis. The samples containing 1.5 to 2.0 μg of total protein were separated on 5% sodium dodecyl sulfate polyacrylamide gels. Preparing gels and electrophoresis were performed as previously described [42]. Western blot analysis was performed using chemiluminescence (GE Healthcare, Little Chalfont, UK) according to the manufacturer's instructions. Primary anti-GFP (Abcam, Cambridge, UK) and anti-rabbit secondary antibody conjugated to horseradish peroxidase (GE Healthcare) were used at 1 4000 and 1 5000 dilution, respectively.

### Conidiation, germination, and pathogenicity assay

To measure conidiation, the same sized agar blocks of the wild type, Δ*myt3*, Δ*myt3*-com, and *MYT3*-oe strains grown on CM were used to inoculate 5 ml of CMC liquid medium. The conidia were counted after 5 days of incubation. To measure germination rate, conidial suspensions (10^6^/ml) of each strain were inoculated into 20 ml of CM and MM including NaNO_3_. The germinated conidia per 100 conidia were counted 0, 2, 4, 8, and 16 h after inoculation. For the pathogenicity assay, 10 μl of conidial suspension (10^6^/ml) obtained from each strain were point-inoculated into a spikelet of the wheat head at early anthesis. Infected plants were incubated in a humidity chamber for 3 days, and subsequently transferred to a green house. After 2 weeks, spikelets showing disease symptoms were counted.

### Quantitative real-time (qRT)-PCR of gene expression

The qRT-PCR was performed using SYBR Green Supermix (Bio-Rad, Hercules, CA, USA) and a 7500 real-time PCR system (Applied Biosystems, Foster city, CA, USA). Each reaction contained 10 μl of SYBR green Supermix, 500 nM of forward and reverse primers, cDNA template, and nuclease-free water to a final volume of 20 μl. PCR cycling conditions were 2 min at 50°C (one cycle); 10 min at 95 °C (one cycle); and 15 s at 95°C followed by 1 min at 60°C (40 cycles). Experiments were repeated two times with three replicates. Expression levels were calculated using the comparative Ct method (Applied Biosystems). *CYP1* gene (FGSG_07439) served as the endogenous reference for normalization.

### Trichothecene analysis

To analyze total trichothecene production, conidial suspensions (10^4^/ml) of each strain were inoculated in MMA, and the cultures were incubated for 7 days at 25°C. The culture media was filtered with Miracloth. Trichothecenes were extracted from 150 μl of culture filtrates by mixing with 250 μl of ethyl acetate/methanol solution (4 1, v/v) [43]. Extracts were dried and derivatized with Sylon BTZ (BSA + TMCS + TMS1, 3 2 3; Supelco, Bellefonte, PA, USA). Sequentially, 200 μl of *n*-hexane and 200 μl of distilled water were added to the reaction products. This reaction was left standing until two layers separated. Trichothecenes in the upper layer were analyzed with a Shimadzu QP-5000 gas chromatograph-mass spectrometer (GC-MS; Shimadzu, Kyoto, Japan) [44]. The total trichothecene concentration was quantified based on the biomass of each strain in MMA.

### Sexual crosses

From strains grown on carrot agar media [29] for 5 days, aerial mycelia were removed with 700 μl of 2.5% Tween 60 solution to induce fertilization. The plates were incubated under a near-UV light for 7 to 10 days. For outcrosses, mycelia of a female strain grown on carrot agar media were fertilized with 1 ml of conidial suspension (10^6^/ml) obtained from male strain, which was induced in CMC. The heterothallic Δ*mat2* mutant, a deletion strain of *MAT1-2*, was used as a tester mutant for outcrosses [45]. Perithecia and ascospores were observed 9 days after sexual crosses.

## Results

### Identification of Myb-like transcription factor MYT3

**Figure 1:**
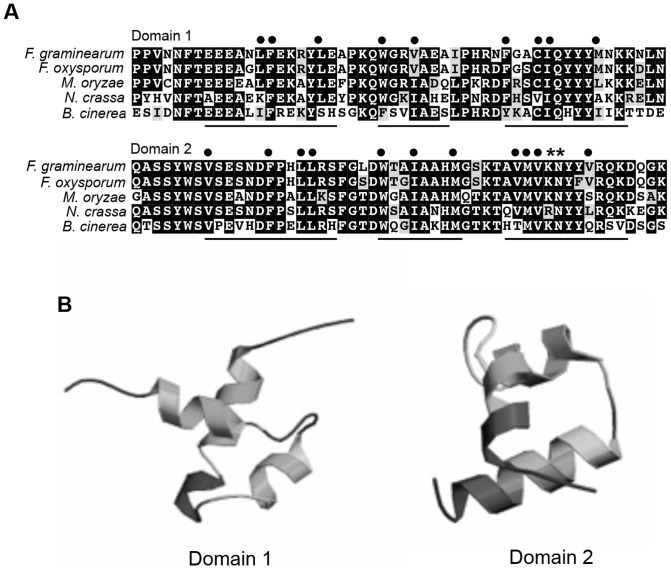
Alignment of Myb-like DNA-binding domains of MYT3 homologs. (A) MYT3 homologs in *Fusarium oxysporum* (FOXG_00743), *Magnaporthe oryzae* (MGG_14558), *Neurospora crassa* (NCU_10346), *Botrytis cinerea* (BC1G_02829) were used for this analysis. Each Myb domain contains three helices, indicated with an underline, and residues that form their hydrophobic cores, indicated by black dots above the residue. The second domain contains residues that are implicated to have direct contact with the DNA, indicated by asterisks [21], [46]. (B) Ribbon diagram of Myb-like DNA binding domains in MYT3.

*MYT3* has an open reading frame (ORF) of 7,340 base pairs (bp) with three introns, and is predicted to encode a 2,304-amino acid protein that contains two Myb-like DNA-binding domains (residues 1,263 to 1,314 and 1,534 to 1,586; Figure 1A). BLAST and phylogenetic analysis of homologous proteins in other fungi demonstrated that MYT3 is more highly conserved within species of the subphylum Pezizomycotina of the Ascomycota than in the phyla Oomycota, Basidiomycota and the subphylum Saccharomycotina (Figure S1A). Additionally, the Myb-like DNA binding domains of MYT3 showed high sequence similarities to *F. oxysporum* (FOXG_00743, 93%), *Magnaporthe oryzae* (MGG_14558, 74%), *Neurospora crassa* (NCU_10346, 69%), and *Botrytis cinerea* (BC1G_02829, 54%) (Figure 1A and S1B) although the other regions of MYT3 homolog proteins were variable. Currently, none of the MYT3 homologs has been functionally characterized. Each Myb-like DNA binding domain of MYT3 consists of 52 amino acids, forming a helix-turn-helix structure with hydrophobic residues (Figure 1A and B). The third helix of the second domain contains residues (KN) that are implicated to be in direct contact with DNA (Figure 1A) [46], suggesting that MYT3 possibly functions in transcriptional regulation.

### MYT3 is consistently expressed in nuclei

**Figure 2:**
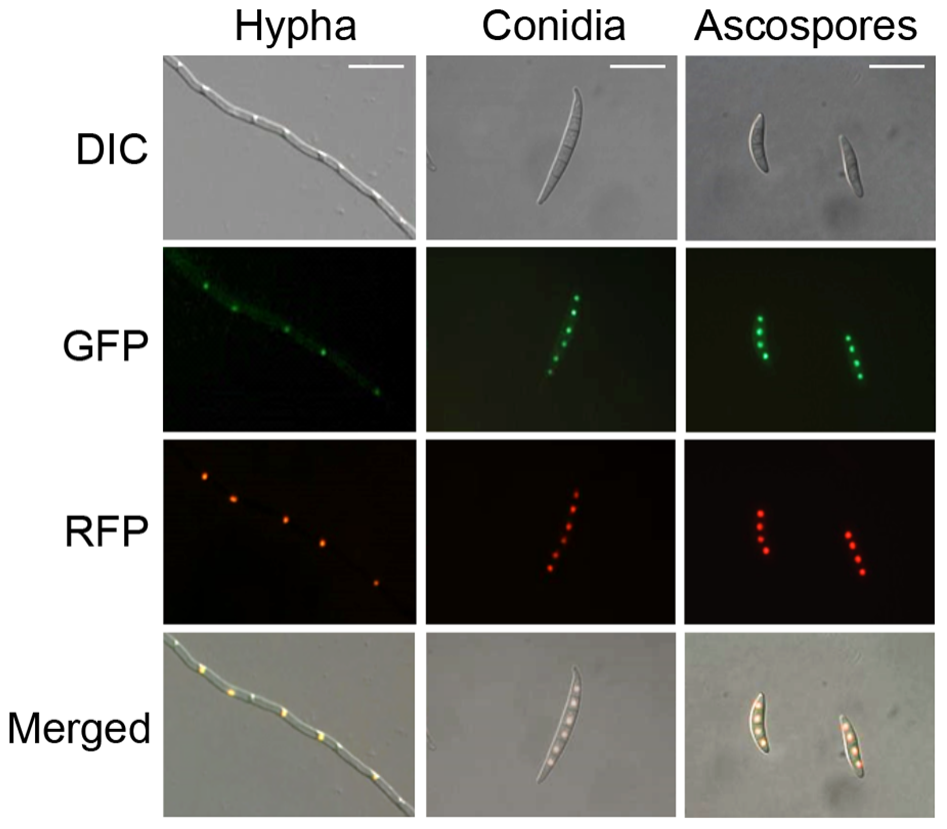
Nuclear localization of MYT3. Representative examples show co-localization of MYT3 fused with green fluorescent protein (GFP) and histone H1 fused with red fluorescent protein (RFP). A strain containing MYT3::GFP and hH1::RFP was grown on CM (hypha), YMA (conidia), and carrot agar media (ascospores) for the microscopic observation, respectively. DIC, differential interference contrast; scale bar = 20 μm.

In addition to the Myb DNA-binding domains, we identified several nuclear localization sequences (NLSs) in MYT3, including mono- and bi-partite sequences (data not shown). Therefore, we next investigated whether MYT3 localizes in the nucleus. To do this, we generated a strain that has GFP fused to C-terminus of MYT3 in the wild-type strain, named by *MYT3*-gfp (). Thirteen transformants containing single *MYT3*::*GFP* copy were obtained, and nuclear GFP signals were observed in these strains. For further confirmation of nuclear localization of MYT3, the *MYT3*-gfp strain was outcrossed with a mat1r strain that contains RFP fused to histone H1 protein in *MAT1-1* deletion background, with expectation to observe co-localization of MYT3::GFP and hH1::RFP in nuclei. From perithecia by this outcrossing, we isolated 20 progeny resistant to both hygromycin and geneticin, and observed that all progeny expressed GFP and RFP in nucleus of the most developmental stages such as hyphae, conidia, and ascospores (Figure 2), indicating that MYT3 is continuously expressed in nuclei.

### Genetic complementation and expression of *MYT3*

**Figure 3:**
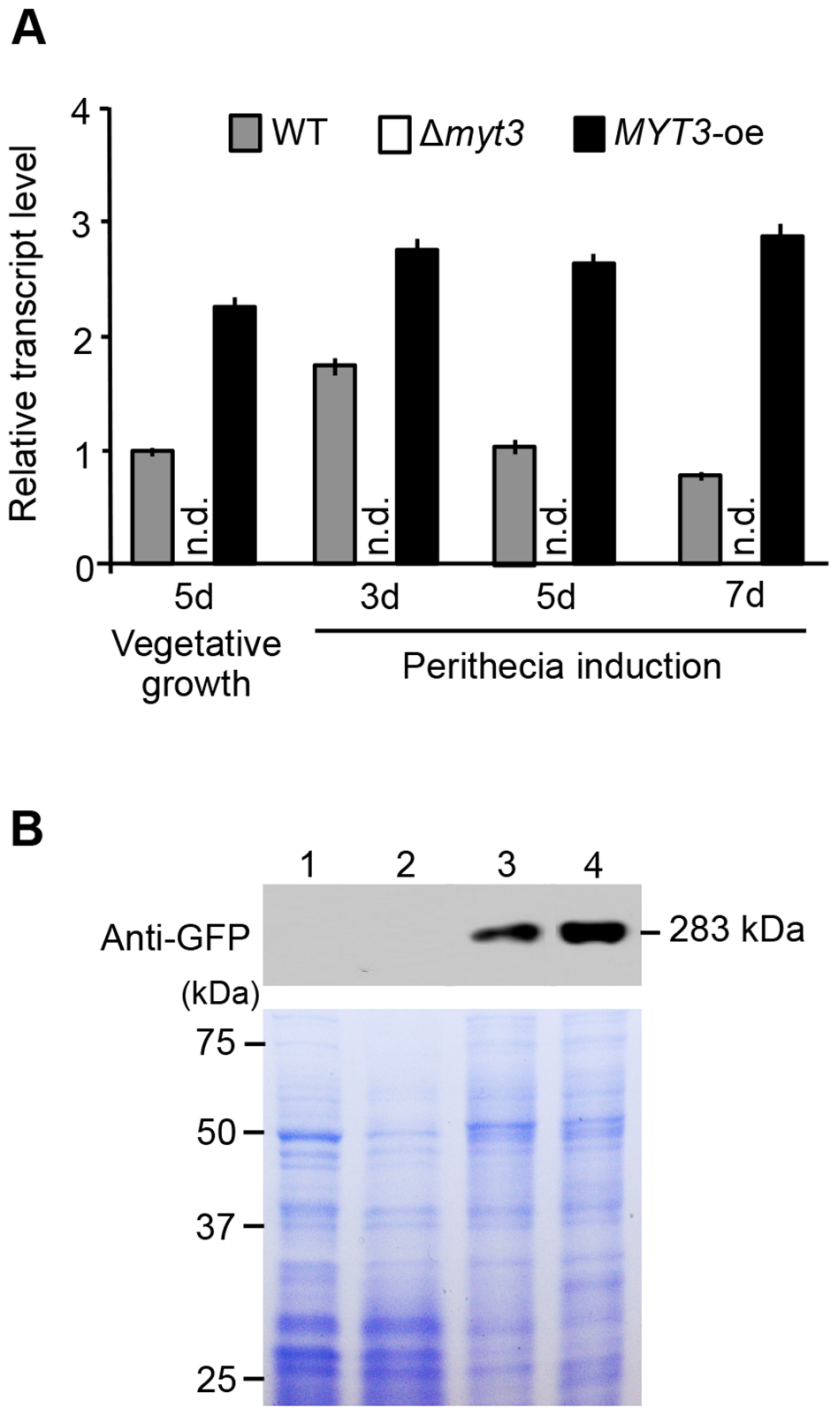
Transcriptional/translational expression of *MYT3*. (A) Transcript levels of *MYT3* in each strain were analyzed by qRT-PCR. Total RNA of each strain was extracted from vegetative cultures grown on carrot agar media, and from sexual cultures 3, 5, and 7 days after induction of sexual development. WT, wild-type strain Z-3639; Δ*myt3, MYT3* deletion mutant; *MYT3*-oe, over expression strain where *MYT3* expression is driven by the *EF1α* promoter. d, day; n.d., not detected. (B) Western blot analysis of *MYT3*-gfp stain. Lane 1, whole cell extracts were prepared from vegetative cultures grown on carrot agar media for 5 days; Lane 2–4, whole cell extracts were from sexual cultures 3, 5, and 7 days after induction of sexual development, respectively. Top, western blotting data; bottom, coomassie blue staining used as a loading control.

To confirm the roles of *MYT3*, we generated a complementation strain, Δ*myt3*-com, by co-transforming the *MYT3* gene and hygromycin resistance gene into the protoplast of the Δ*myt3* strain (Figure S3A). Southern blot analysis indicated that the resulting mutant, designated strain Δ*myt3*-com, obtains a 2.3-kb band of hybridization present in the wild-type strain (Figure S3A). To further dissect the molecular mechanism of the function of *MYT3*, overexpression of *MYT3* was achieved with a construct containing the *EF1α* promoter fused to the *MYT3* ORF (Figure S3B). Strain *MYT3*-oe, obtained by insertion of the constitutive expression construct into the wild type, was confirmed by Southern blot analysis (Figure S3B), and the expression level of *MYT3* in the *MYT3*-oe strain was measured by qRT-PCR (Figure 3A).

Expression of *MYT3* in the *MYT3*-oe strain was consistent at all stages of development, with approximately two- to three-fold higher expression than that of the wild-type strain (Figure 3A). The expression level of *MYT3* in the wild-type strain was slightly increased at 3 days after sexual induction, and decreased over time to the similar level of expression in vegetative growth (Figure 3A). At translational level, the expected 283-kDa protein (MYT3::GFP) in a *MYT3*-gfp strain was detected by anti-GFP (Figure 3B). However, expression of MYT3 was high during sexual development with the highest level at 7 days after sexual induction (Figure 3B). Taken together, these results suggest that *MYT3* play an important role during sexual development.

### Deletion of *MYT3* affects fungal development in response to nitrogen

**Figure 4:**
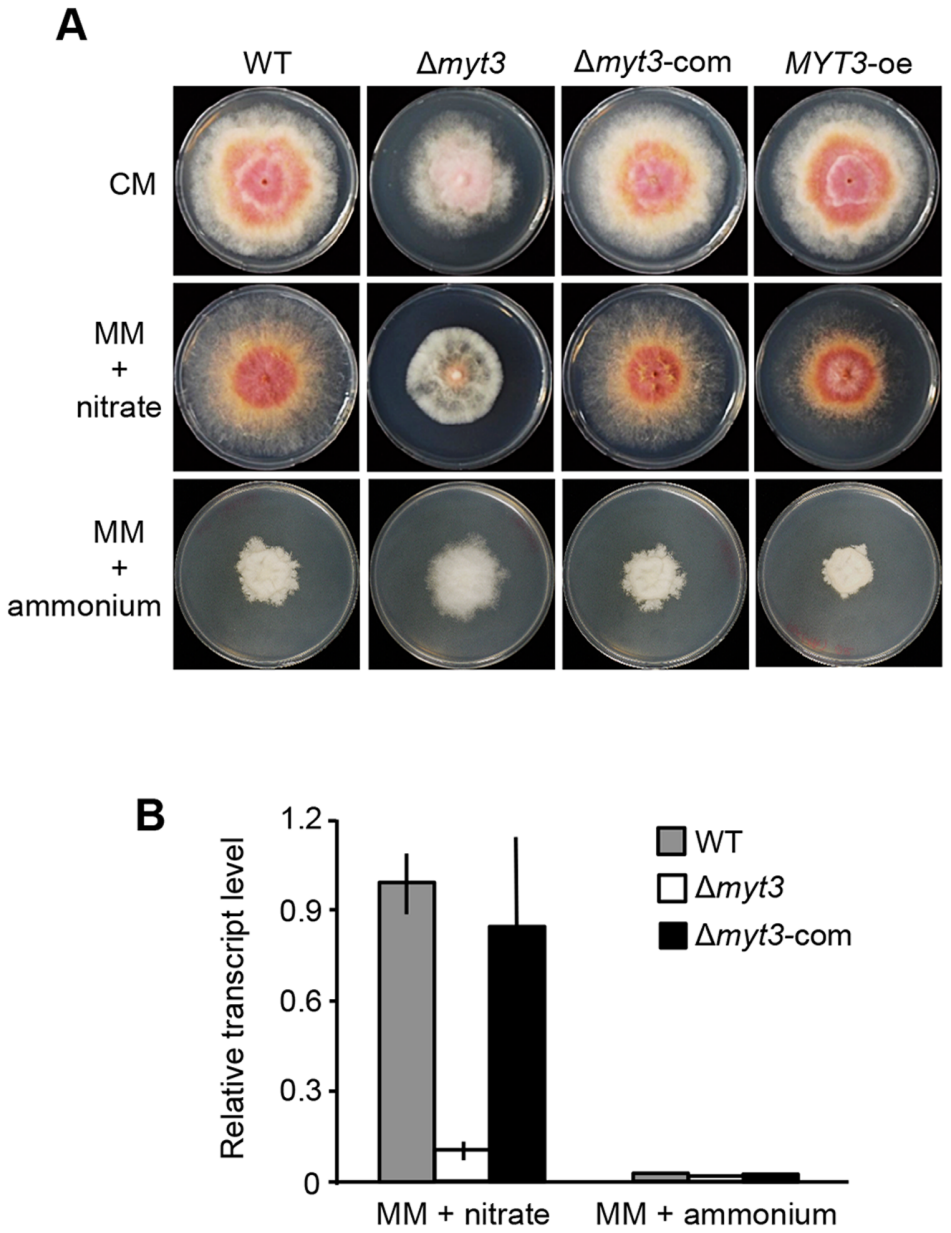
Effects of nitrogen source on fungal growth and expression of nitrate reductase gene. (A) Vegetative growth of each strain on complete media (CM), minimal media (MM) containing nitrate, and MM containing ammonium. Photographs were taken after 5 days (CM, MM + nitrate) and 7 days (MM + ammonium) of incubation. WT, wild-type strain Z-3639; Δ*myt3, MYT3* deletion mutant; Δ*myt3*-com, complemented strain of the Δ*myt3*; *MYT3*-oe, overexpressed strain of *MYT3*. (B) To measure expression of nitrate reductase gene, all strains were grown in CM media for 48 h and then washed with water. The resulting mycelia were transferred into MM+ nitrate or MM + ammonium, and total RNA was extracted 3 h after resuspension.

The deletion mutant of *MYT3* exhibited a growth defect on CM in comparison to the wild type, Δ*myt3*-com, and *MYT3*-oe strains (Figure 4A and Table 2). Also, the Δ*myt3* strain poorly grew on MM lacking nitrogen in comparison to the growth on CM (Table 2). With these observations, increasing expression of ESTs, corresponding to FGSG_00324 (*MYT3*), in nitrogen starvation condition (MIPS, http://www.plexdb.org/) [47] gave rise to a question whether nutrient such as nitrogen sources affects fungal growth of Δ*myt3* strain. On MM containing nitrate, the mutant grew poorly compared to the wild type, whereas fungal growth of the Δ*myt3*-com and *MYT3*-oe strains was similar to that of the wild type (Table 2). When MM was supplemented with ammonium instead of nitrate as a nitrogen source, the Δ*myt3* strain grew better than the wild type, Δ*myt3*-com, and *MYT3*-oe strains (Figure 4A). Additionally, expression level of nitrate reductase gene (FGSG_01947) homologous to *niaD* of *F. fujikuroi* [48] was significantly decreased in the Δ*myt3* strain grown in MM + nitrate (Figure 4B). Although the expression of this gene from the Δ*myt3* strain grown in MM + ammonium was slightly decreased in comparison to the wild type and Δ*myt3*-com, the magnitude of the induction was comparably low in all strains grown MM + ammonium compared to the MM + nitrate (Figure 4B). Together, these results indicate that MYT3 is involved in nitrogen metabolic pathway by regulating the expression of nitrate reductase gene.

**Table 2:** Conidiation, growth, and virulence of *Fusarium graminearum* strains.

		Fungal growth			
Strain	Conidiation (×10^5^ spores/ml)	CM	MM	MM (-N)^a^	Disease index^b^
wild type	16.7±2.2A^c^	76±0.5A	79±1.7A	57±2.1A	11.0±2.2A
Δ*myt3*	0.3±0.2B	61±2.4B	44±1.1B	25±3.1B	1.3±0.5B
Δ*myt3*-com	16.1±1.3A	75±1.3A	77±1.4A	59±2.3A	10.3±2.1A
*MYT3*-oe	17.8±2.2A	76±0.6A	70±1.7C	58±0.6A	12.0±4.5A

a (-N) indicates lacking of nitrogen source in MM.

b Mean and standard deviation of diseased spikelets per wheat head.

c Values with different letters within a column indicate statistical difference (*p*<0.05) based on Tukey's test. All experiments were repeated three times with three replicates each.

**Figure 5:**
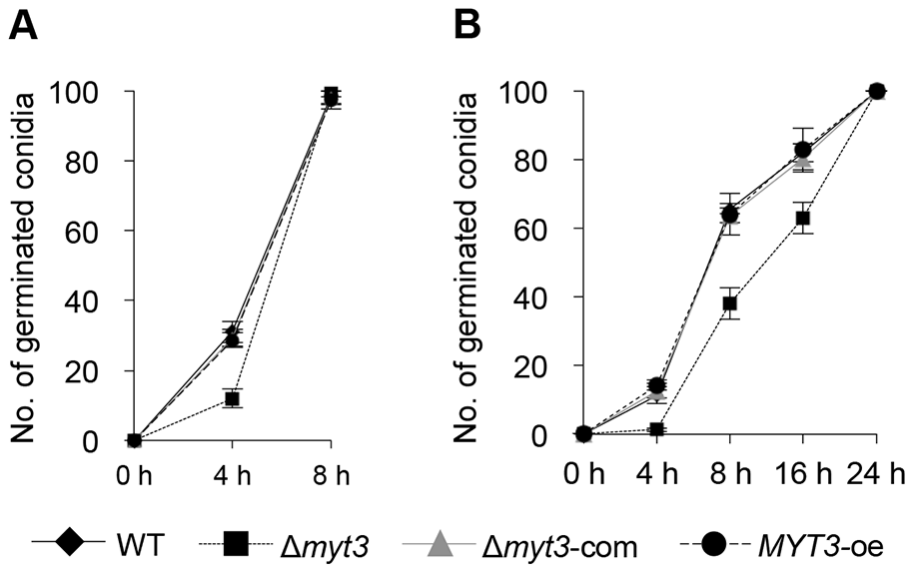
Germination rate of *F. graminearum* strains. (A) Numbers of germinated conidia in CM were counted 0, 4, and 8 h after inoculation. (B) Numbers of germinated conidia on MM containing nitrate were counted 0, 4, 8, 16 and 24 h after inoculation. Each experiment was performed in triplicate two independent times. WT, wild-type strain Z-3639; Δ*myt3, MYT3* deletion mutant; Δ*myt3*-com, complemented strain of the Δ*myt3*; *MYT3*-oe, overexpressed strain of *MYT3*.

The Δ*myt3* strain produced approximately 50-fold less conidia compared to the wild type 5 days after inoculation into CMC media, but there was no significant difference in conidiation among the wild type, Δ*myt3*-com, and *MYT3*-oe strains (Table 2). In addition, the germination rate of the Δ*myt3* strain on CM (Figure 5A) and MM including nitrate (Figure 5B) was markedly slower than that of other strains. Taken together, these results represent that deletion of *MYT3* contributes to the impairment of fungal development and growth.

### *MYT3* is involved in pathogenicity during infection of wheat

**Figure 6:**
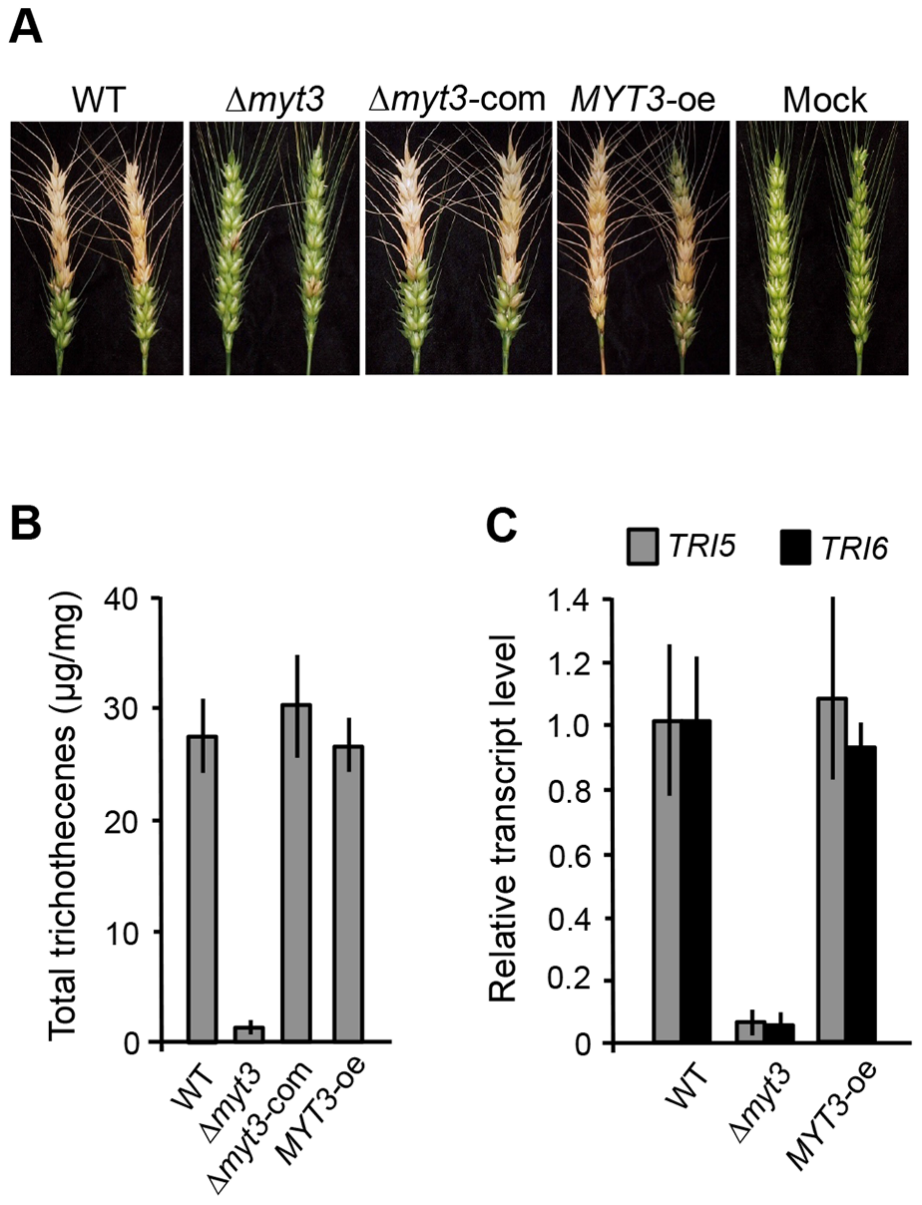
Virulence assay and trichothecene analysis. (A) Flowering wheat head were inoculated with conidia of the wild-type (WT) Z-3639, Δ*myt3* mutant, complemented strain Δ*myt3*-com, and overexpressed strain *MYT3*-oe, respectively. Mock indicates a negative control inoculated with 0.01% Tween 20. Wheat heads were photographed 14 days after inoculation. (B) Analysis of total trichothecenes (deoxynivalenole and 15-acetyl-deoxynivalenol) was performed on each strain grown in minimal liquid medium supplemented with 5 mM agmatine (MMA). The measured amount of trichothecenes was normalized to the biomass of each strain. (C) Expression of *TRI5* and *TRI6* in the wild type, Δ*myt3*, *MYT3*-oe strains. Transcript levels were analyzed by qRT-PCR from cultures grown in MMA for 4 days and presented as relative transcript level compared to a control gene, *CYP1*.

Our observations that the Δ*myt3* strain grows slowly on CM and MM (Table 2) led us to question whether the deletion of *MYT3* affects *F. graminearum* pathogenicity during wheat infection. To evaluate pathogenicity on flowering wheat heads, conidial suspensions of each strain were point-inoculated on a spikelet and incubated in a greenhouse. The wild-type strain induced normal head blight symptoms, which manifests as discoloration, at 14 days after inoculation (Figure 6A). In contrast, the Δ*myt3* strain was restricted to infection sites, which were unable to spread from the rachis to reach adjacent spikelets on the head (Figure 6A). Consequently, the average disease index of the Δ*myt3* strain was approximately 10-fold lower than the wild-type level (Table 2). The infections by Δ*myt3*-com and *MYT3*-oe strains displayed the similar levels of pathogenicity to the wild-type strain (Figure 6A and Table 2). Thus, our results reveal that MYT3 plays an important role in the progression of head blight after initial colonization.

### *MYT3* has an effect on the biosynthesis of trichothecenes

To determine whether *MYT3* affects trichothecene biosynthesis, we measured trichothecene production by the wild type, Δ*myt3*, Δ*myt3*-com, and *MYT3*-oe strains grown on MMA. After normalizing to the amount of fungal biomass, the Δ*myt3* strain produced approximately 10-fold less trichothecenes compared to the wild type (Figure 6B). Furthermore, the ability to produce trichothecenes was restored in the Δ*myt3*-com strain (Figure 6B). To confirm this observation, we investigated the expression of the trichothecene synthesis genes *TRI5* (FGSG_03537) and *TRI6* (FGSG_03536), which encode a trichodiene synthase and a major transcriptional regulator for trichothecene production, respectively [49]. In the Δ*myt3* strain, the expression levels of *TRI5* and *TRI6* were reduced more than six-fold (Figure 6C) compared to the wild type and *MYT3*-oe strains. Together, these results show that the reduction in trichothecene biosynthesis is attributable to disruption of *MYT3*.

### *MYT3* is dispensable for male/female fertility in outcrosses

**Figure 7:**
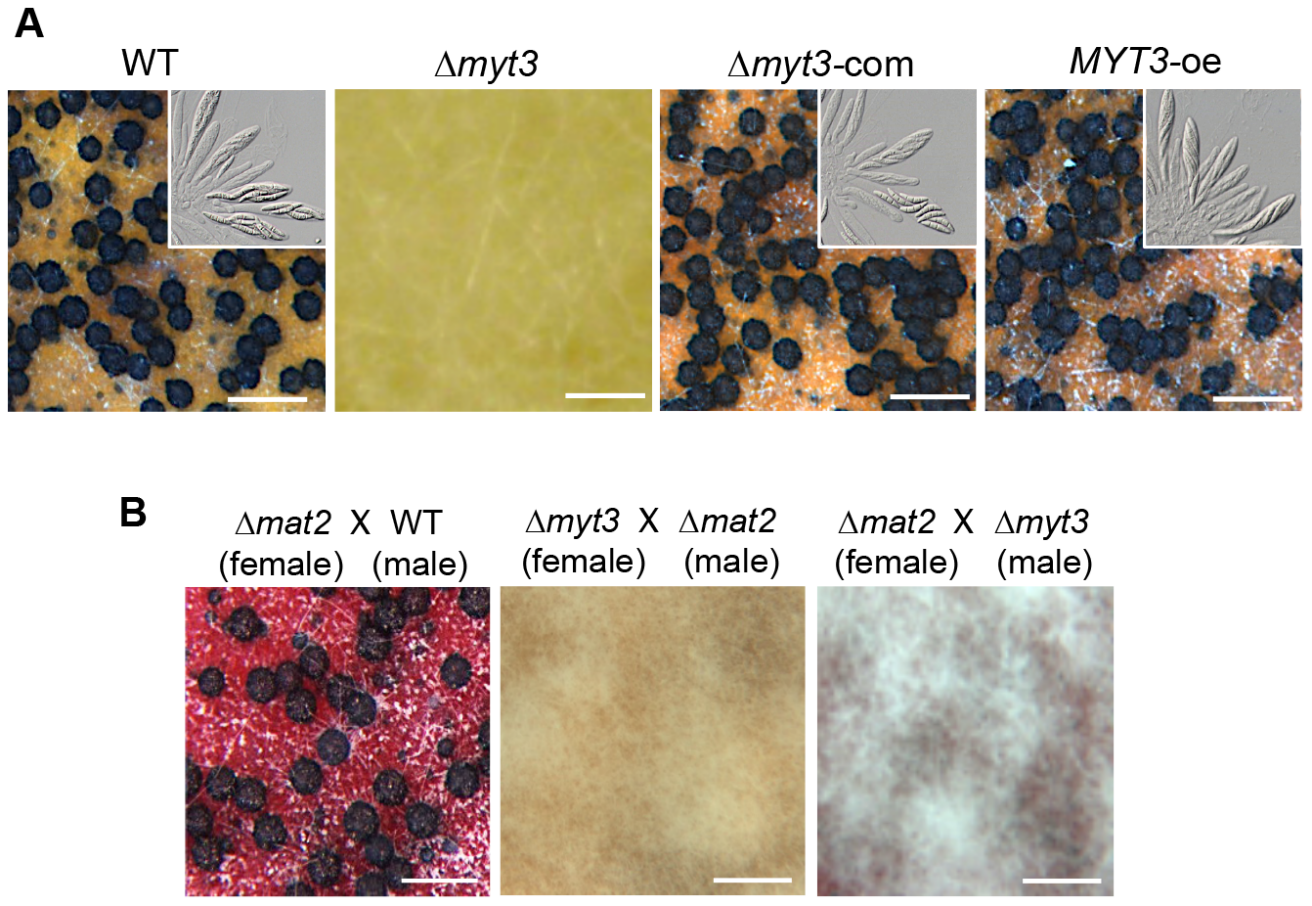
The Δ*myt3* strain exhibits a lack of sexual development. (A) Strains grown on carrot agar media were self-fertilized. Photos were taken 9 days after sexual induction. Dissecting the perithecia showed the asci and ascospores of each strain (inset boxes). WT, wild-type strain Z-3639; Δ*myt3, MYT3* deletion mutant; Δ*myt3*-com, complemented strain of the Δ*myt3*; *MYT3*-oe, overexpressed strain of *MYT3*. Scale bar = 500 μm. (B) Heterozygous outcrosses. The Δ*myt3* strain, as either a female or male strain, was outcrossed with self-sterile Δ*mat2* strain. Photos were taken 9 days after outcrossing. Scale bar = 500 μm.

Self-fertility of the Δ*myt3* strain was determined from the formation of perithecia and ascospores on carrot agar media. The wild-type strain began to produce protoperithecia 3 days after sexual induction. After an additional 3- or 4-day incubation, mature perithecia were formed; the perithecia contained asci, each with eight ascospores (Figure 7A). In contrast, the Δ*myt3* strain only produced mycelia and/or conidia, and never produced fruiting bodies (Figure 7A). The sexual defects in the Δ*myt3* strain were recovered in the Δ*myt3*-com strain.

When a conidial suspension of the Δ*myt3* strain was spermatized to a self-sterile Δ*mat2* strain, a deletion mutant of *MAT1-2*, no perithecia were formed on the outcrossed cultures, indicating that the Δ*myt3* strain lost male fertility (Figure 7B). Furthermore, when the Δ*myt3* strain was used as a female strain in outcrossing with the Δ*mat2* strain, no perithecia were found on the carrot agar media (Figure 7B). Taken together, these results show that deletion of *MYT3* abolishes both male and female fertility.

## Discussion

Previously, we reported that the Myb-like proteins MYT1 and MYT2 play important roles in female fertility and perithecium size during the sexual development of *F. graminearum* [12], [13]. Specifically, deleting *MYT2* enhanced vegetative growth and increased the number of ascospores [13]. In addition, we observed that MYT2 displays differential expression in germinated hyphae compared to ascospores and conidia [13]. These results suggest that *MYT2* suppresses cell proliferation in various developmental stages, as well as critical roles for Myb-like proteins. Except with *MYT1* and *MYT2*, the *F. graminearum* genome has several Myb-like proteins whose biological functions remain unclear. As a series of Myb-like proteins in *F. graminearum*, we characterized the roles of *MYT3* in aspects of fungal development and pathogenicity.

The Myb proteins belong to a large family of transcription factors that are essential for regulating genes for cellular processes. Extensive studies of Myb-like proteins in various eukaryotes have demonstrated that the roles of Myb proteins are functionally diverse. Myb proteins are involved in proliferation, differentiation, metabolic pathways, cell fate, and stress responses [15], [16], [17]. In this study, we found that MYT3 contains two Myb-like DNA binding domains, which are likely to build a helix-turn-helix structure with hydrophobic residues and conserved in the subphylum Pezizomycotina. However, due to the low sequence similarity with comparison of other Myb proteins identified previously, we cannot conclude whether MYT3 belongs to a certain subfamily of Myb proteins (e.g., R1, R2R3, or R1R2R3). Myb proteins have diverged substantially between organisms. For examples, 4R-Myb subfamily of Myb proteins was identified in plants, and consists of four repeats; one of the repeats contain R1/R2-like repeat [14]. The oomycetes *P. infestans* has an atypical class of Myb proteins that contain multi-domains, and neither of the tandem Myb domains resembles the R2 or R3 region of c-Myb. Additionally, several proteins with novel combinations of Myb domains were observed in this organism [21]. The *F. graminearum* genome is different from other eukaryotes in the total number of Myb proteins. The *F. graminearum* genome was reported to contain 19 Myb-like proteins [11], whereas *Arabidopsis thaliana*, *Oryzae sativa*, and *P. sojae* were predicted to express 198, 183, and 68 Myb-like proteins, respectively [28], [50]. In addition, most Myb proteins of *F. graminearum* have either one or two Myb-like DNA binding domains without the presence of a signature sequence, such as SHAQKY (data not shown) [51]. Taken together, Myb-like proteins in *F. graminearum* seem to be highly divergent within this species.

The regulatory functions of Myb proteins in response to nutritional stimuli, such as nitrogen and carbon, have been studied in other fungi. The deletion mutant of *flbD*, a Myb transcription factor, in *A. nidulans* showed an impaired ability to produce conidia under nitrogen starvation conditions [26]. In *Schizosaccharomyces pombe*, the Myb-type DNA binding protein Reb1 regulates the transcription of the *ste9+* gene by binding its promoter, which is involved in G1 cell cycle arrest and sexual differentiation in response to nitrogen starvation [52]. Similar to the fungi, it was reported that CmMYB1, an R2R3-type MYB transcription factor, is responsible for the expression of nitrogen assimilation genes in a unicellular red alga *Cyanidioschyzon merolae* [53]. *CmMYB1* expression was induced by nitrogen depletion, and the *CmMYB1* mutant showed decreased cell viability after exposure to nitrogen-depleted conditions compared to the parental strain [53]. CmMYB1 specifically occupies the promoters of nitrogen assimilation genes only under nitrogen-depleted conditions, indicating that CmMYB1 is a central nitrogen regulator in *C. merolae* [53].

Related with the regulation of nitrogen metabolism, the mechanism mediated by global regulators AreA/Nit2 has been extensively studied in filamentous fungi [54], [55], [56]. The transcriptional regulator AreA activates the expression of nitrogen assimilatory genes such as nitrate reductase gene by binding its promoter when favored nitrogen sources, such as glutamine and ammonium, are not available [57], [58]. In this study, with our observations that growth defect of the Δ*myt3* strain on MM and MM containing nitrate as a nitrogen source, we measured that expression of *FgniaD*, which encodes a nitrate reductase, was decreased in the Δ*myt3* strain grown on MM + nitrate compared to the wild type. These observations gave rise to the hypothesis that MYT3 is involved in nitrogen assimilatory pathway. This hypothesis is supported by our observation that *FgniaD* has two putative Myb DNA-binding sequences at −47 and −227 bp upstream of the start codon, based on the binding sequences of 5′-Y(T/C)AACK(G/T)G-3′ as previously described [46]. This binding motif was shown to be responding to the Lys1575 and Asn1576 residues in the second Myb-like domain of MYT3, which has been implicated to participate in direct contact with DNA [46], [59]. However, further studies are needed to clarify whether Myb DNA-binding domains of MYT3 in *F. graminearum* bind to the *FgniaD* promoter.

Considering that MYT3 is conserved in species of the subphylum Pezizomycotina of the Ascomycota, and that transcriptional/translational expression of *MYT3* is increased during sexual development, we hypothesized that MYT3 plays an important role in sexual development. Most *F. graminearum* deletion mutants that have a sexual development defect exhibit an impairment in female fertility, but retain male fertility; for example, *ZIF1* (b-ZIP transcription factor), *FgVelB* (velvet regulator), and *FgflbA* (regulator of G protein signaling) [7], [60], [61]. A *MYT1* deletion mutant also resulted in a defect in female fertility, but retained male fertility [12]. Interestingly, the *MYT3* deletion mutant lost both female and male fertility, and a complemented strain recovered all of the defects. To dissect these observations, we investigated whether deletion of *MYT3* has an effect on vegetative compatibility. In this study, we generated *nit1* and *nitM* mutants derived from the wild type and Δ*myt3* strains (data not shown), respectively, and tested their ability to form heterokaryon by hyphal fusion from the pairing combinations (WT *nitM* × Δ*myt3 nit1*, WT *nit1* × Δ*myt3 nitM*, and Δ*myt3 nit1* × Δ*myt3 nitM*). However, all combinations produced fungal colonies (data not shown), indicating that the *nit* strains used in this study were in same vegetative compatibility group, and deletion of *MYT3* did not cause vegetative incompatibility. Thus, these results suggest that *MYT3* is not involved in heterokaryon formation through hyphal fusion.

We demonstrated that *MYT3* plays an important role in *F. graminearum* on wheat heads. As previously mentioned, *PsMYB1* is also involved in *P. sojae* pathogenesis of soybean, which silencing of *PsMYB1* resulted in the impairment of zoosporogenesis and zoospore development [28]. In plants, several Myb transcription factors (e.g., *AtMYB30*, *TaPIMP1*, *JAmyb*, and *BOS1*) are involved in host resistance to pathogens and abiotic stress, which involves signaling pathways for plant defense molecules, such as jasmonate, salicylic acid, and abscisic acid [28], [62], [63], [64]. Our results also showed that the disease index of the Δ*myt3* strain was approximately 10-fold less than the wild-type level, resulting from an inability of the Δ*myt3* strain to spread from an inoculated spikelet to other spikelets. In addition, trichothecene production and *TRI5* and *TRI6* gene transcript levels were remarkably reduced in the Δ*myt3* strain. This finding may also contribute to the reduced progression of plant disease because trichothecenes are an important virulence factor in *F. graminearum* [65]. Furthermore, we cannot exclude the possibility that MYT3 is required to respond to an environmental cue from the host that helps regulate pathogenesis: this possibility is supported by our observations of different growth pattern of the Δ*myt3* strain to specific nitrogen source. Thus, considering involvement of MYT3 in pathogenesis, identification of target genes regulated by MYT3 will contribute critical information to help understand regulatory mechanisms of *F. graminearum* pathogenesis.

In summary, we examined the biological functions of MYT3, a Myb-like protein in *F. graminearum* that contains two Myb DNA-binding domains. Our results showed that MYT3 is localized in nuclei during developmental stages, affects various aspects of fungal development, such as conidiation, germination, and male/female fertility, and contributes to pathogenicity by either directly or indirectly influencing trichothecene biosynthesis. Our identification of these functions for *MYT3* sheds further light on the complex roles of Myb-like proteins in fungal development and pathogenicity of *F. graminearum*.

## Supporting Information

Figure S1 **Distribution and phylogenetic analysis of MYT3 homologs in fungi.**
(PDF)

Figure S2 **Strategy for fusion of** ***GFP*** **to** ***MYT3*** **.**
(PDF)

Figure S3 **Strategies for complementation and overexpression of** ***MYT3*** **.**
(PDF)

Table S1 **Primers used in this study.**
(PDF)
