# Supplementary material for: MYT3, A Myb-Like Transcription Factor, Affects Fungal Development and Pathogenicity of Fusarium graminearum
Source: PLoS One. 2014 Apr 10;9(4):e94359. doi: 10.1371/journal.pone.0094359 (PMC3983115; doi:10.1371/journal.pone.0094359)
Supplement: Figure S1 — Distribution and phylogenetic analysis of MYT3 homologs in fungi. (PDF) [file pone.0094359.s001.pdf]

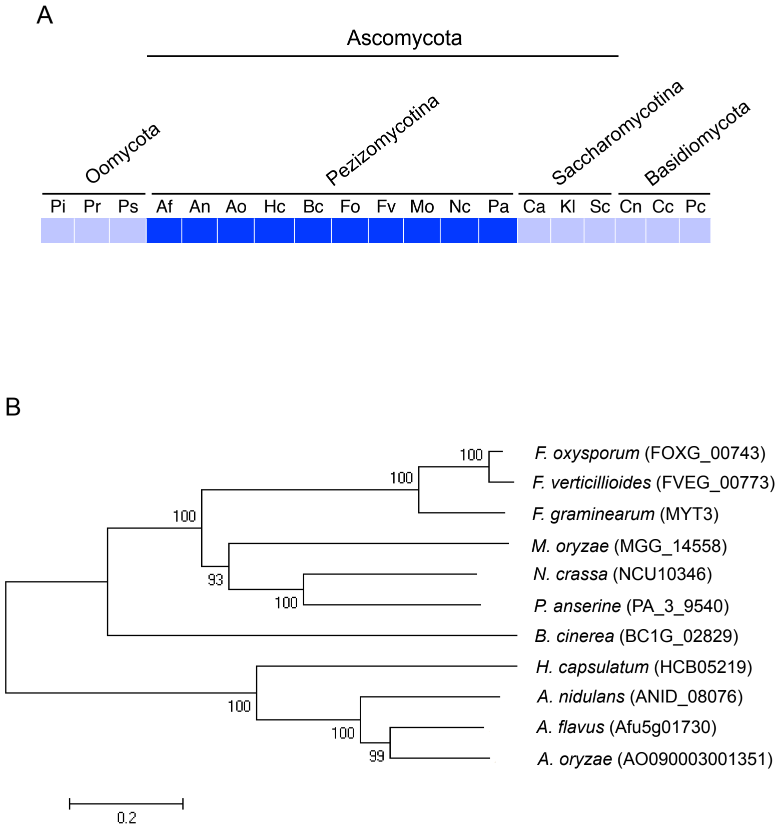

Figure S1 Distribution and phylogenetic analysis of MYT3 homologs in fungi. (A) Distribution of MYT3 in representative fungal species. The BLASTMatrix tool on the Comparative Fungal Genomics Platform (http://cfgp.riceblast.snu.ac.kr/) was used to obtain the distribution image (Park et al. 2008). (B) Phylogenetic tree of MYT3 homologs in several fungal species. Alignment was performed with Clustal Omega (Sievers et al. 2011), and the MEGA program (Tamura et al. 2011) was used to perform a 1,000-bootstrap phylogenetic analysis using the neighbor-joining method. Pi, *Phytophthora infestans*; Pr, *P. ramorum*; Ps, *P. sojae*; Af, *Aspergillus fumigatus*; An, *Aspergillus nidulans*; Ao, *Aspergillus oryzae*; Hc, *Histoplasma capsulatum*; Bc, *Botrytis cinerea*; Fo, *Fusarium oxysporum*; Fv, *Fusarium verticillioides*; Mo, *Magnaporthe oryzae*; Nc, *Neurospora crassa*; Pa, *Podospora anserine*; Ca, *Candida albicans*; Kl, *Kluyveromyces lactis*; Sc, *Saccharomyces cerevisiae*; Cn, *Cryptococcus neoformans*; Cc, *Coprinus cinereus*; Pc, *Phanerochaete chrysosporium*.

Supplementary references

Park J, Park B, Jung K, Jang S, Yu K, et al. (2008) CFGP: a web-based, comparative fungal genomics platform. Nucleic Acids Res 36: D562–D571.

Sievers F, Wilm A, Dineen DG, Gibson TJ, Karplus K, et al. (2011) Fast, scalable generation of high-quality protein multiple sequence alignments using Clustal Omega. Mol Syst Biol 7: 539.

Tamura K, Peterson D, Peterson N, Stecher G, Nei M, et al. (2011) MEGA5: Molecular Evolutionary Genetics Analysis using Maximum Likelihood, Evolutionary Distance, and Maximum Parsimony Methods. Mol Biol Evol 28: 2731–2739.
