# Supplementary material for: MYT3, A Myb-Like Transcription Factor, Affects Fungal Development and Pathogenicity of Fusarium graminearum
Source: PLoS One. 2014 Apr 10;9(4):e94359. doi: 10.1371/journal.pone.0094359 (PMC3983115; doi:10.1371/journal.pone.0094359)
Supplement: Figure S2 — Strategy for fusion of GFP to MYT3 . (PDF) [file pone.0094359.s002.pdf]

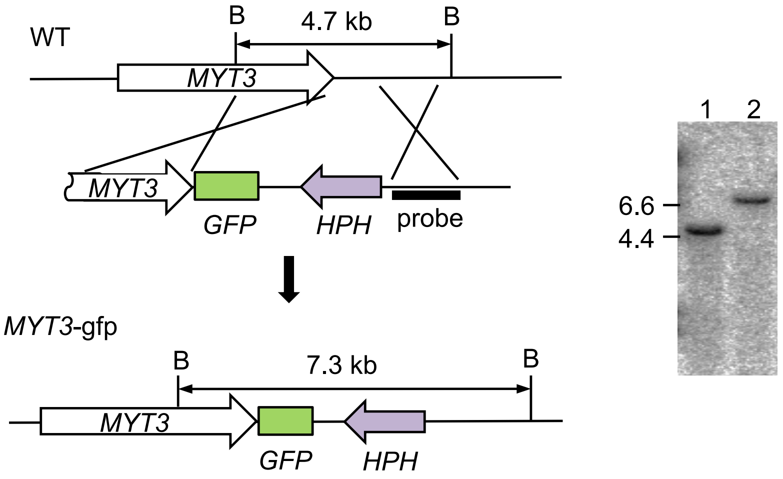

Figure S2 Schematic illustrating the strategy for fusion of *GFP* to *MYT3*. Left panel: Strategy used to fuse *GFP* to the 3′ end of *MYT3*. Right panel: Southern blot analysis confirming genetic construct. Lane 1, wild-type strain; Lane 2, *MYT3*::*GFP* strain (*MYT3*-gfp). Sizes of the DNA standards (kb) are indicated to the left of the blot. B, *Bgl*II.
