# Supplementary material for: MYT3, A Myb-Like Transcription Factor, Affects Fungal Development and Pathogenicity of Fusarium graminearum
Source: PLoS One. 2014 Apr 10;9(4):e94359. doi: 10.1371/journal.pone.0094359 (PMC3983115; doi:10.1371/journal.pone.0094359)
Supplement: Figure S3 — Strategies for complementation and overexpression of MYT3 . (PDF) [file pone.0094359.s003.pdf]

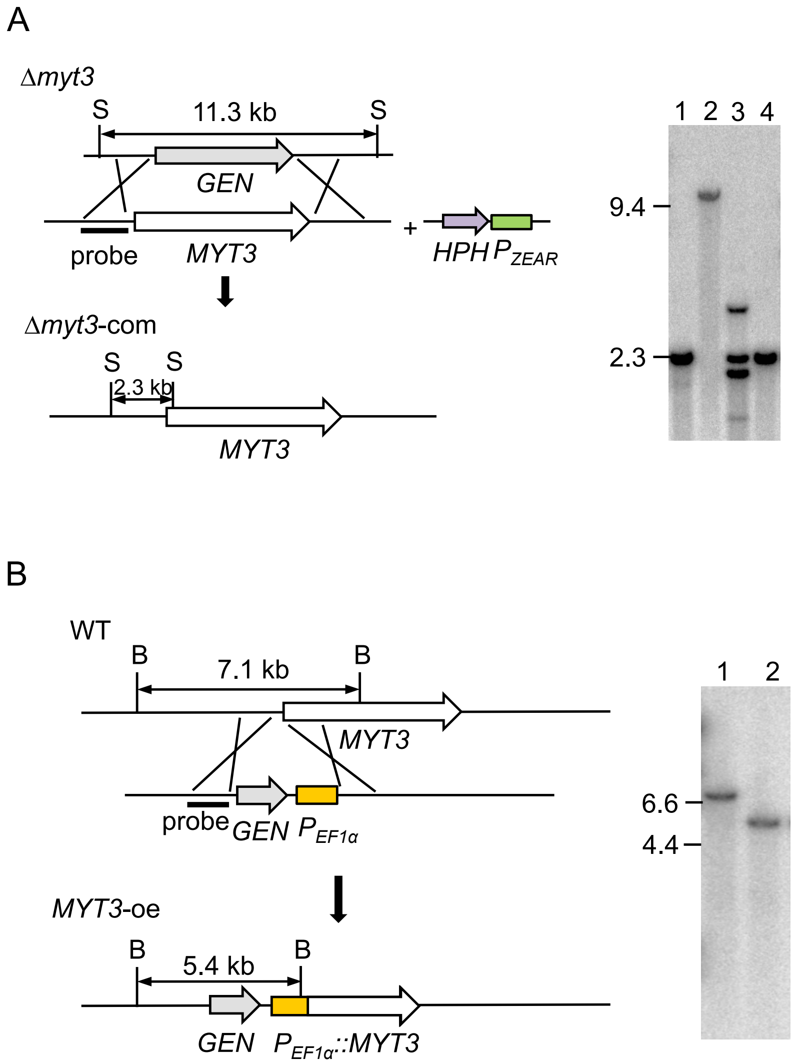

Figure S3 Strategies for complementation and overexpression of *MYT3*. (A) Left panel: schematic drawing illustrating the construction of the complemented strain. The Δ*myt3*-com strain was derived from the Δ*myt3* strain. Two PCR fragments including *MYT3* ORF and *HPH* were co-transformed into the *Δmyt3* protoplast. Right panel: Southern blot analysis confirming genetic constructs. Lane 1, wild-type strain; Lane 2, Δ*myt3* strain; Lane 3, ectopic transformant of Δ*myt3* strain; Lane 4, complemented strain (Δ*myt3*-com). The sizes of the DNA standards (kb) are indicated to the left of the blot. S, *Sal*I. (B) Left panel: schematic drawing illustrating the strategy to generate an overexpression strain. The cassette containing *EF1α* promoter and geneticin resistance gene was substituted for the *MYT3* promoter region. Right panel: Southern blot analysis confirming genetic constructs. Lane 1, wild-type strain; Lane 2, *MYT3*-overexpressed strain (*MYT3*-oe) derived from the wild-type strain. Sizes of the DNA standards (kb) are indicated to the left of the blot. B, *Bgl*II.
