# Supplementary material for: MYT3, A Myb-Like Transcription Factor, Affects Fungal Development and Pathogenicity of Fusarium graminearum
Source: PLoS One. 2014 Apr 10;9(4):e94359. doi: 10.1371/journal.pone.0094359 (PMC3983115; doi:10.1371/journal.pone.0094359)
Supplement: Table S1 — Primers used in this study. (PDF) [file pone.0094359.s004.pdf]

Table S1. Primers used in this study

Primer	Sequence (5′→3′)
MYT3-5N	ATCCTCCTTCCCGATGTCCTTAGCA
MYT3-3N	AACATCGGCCCTGAGAACCCTAC
HPH-F	CAACAGAGTGAACAGCGCCGATAC
HPH-R	TGGAGGCTGGGCAAAAGACGA
MYT3-5F	GACACCACAGCCTCATCATTACGA
MYT3-5R	GAACAGCTCCTCGCCCTTGCTCACTCTTCTACTATAAGGATCCGGATGTCTC
GFP-F	GTGAGCAAGGGCGAGGAGCTG
HYG-F1	GGCTTGGCTGGAGCTAGTGGAGG
MYT3-3F	CCTCCACTAGCTCCAGCCAAGCCCAGCATTTGTCGTTTTGTTAGGTCC
MYT3-3R	ACTGAACGCGAGGAGAACATTGAC
MYT3-gfpF	CGCCTACTAGCCGTCCTGTGTC
MYT3-gfpR	AACATCGGCCCTGAGAACCCTAC
UP-5F	CGCAGATGGTACCCCCGAATAG
5R-GEN	GATAGTGGAAACCGACGCCCCCGCTTAGTGGTGGATGAGAAGGG
Neo-F	GGGGCGTCGGTTTCCACTATC
EF-R	CTTTGAAGATTGGGTTCCTTTTGTGATA
MYT3-F	TATCACAAAAGGAACCCAATCTTCAAAGGCAACAATGGAGTCCCAGTCG
MYT3-R	CCCATACCCATAGGAGGCATAGC
MYT3-RN	GGCAGCGCTGGCAGAAACATA
CYP-rtF	TCAAGCTCAAGCACACCAAGAAGG
CYP-rtR	GGTCCGCCGCTCCAGTCT
MYT3-rtF	CACCCCTCCACCGCAAACTAAC
MYT3-rtR	AAGTTGGCTTGAGAGGTGGCATTC
TRI5-rtF	GCCATTTTGGACCTTTCTGCTCATT
TRI5-rtR	GCCATAGAGAAGCCCCAACACAAT
TRI6-rtF	GGCAACCATTCAAGCGCTTTTTCT
TRI6-rtR	CACCCTGCTAAAGACCCTCAGACATT
